# Atlantic Sturgeon Spatial and Temporal Distribution in Minas Passage, Nova Scotia, Canada, a Region of Future Tidal Energy Extraction

**DOI:** 10.1371/journal.pone.0158387

**Published:** 2016-07-06

**Authors:** Michael J. W. Stokesbury, Laura M. Logan-Chesney, Montana F. McLean, Colin F. Buhariwalla, Anna M. Redden, Jeffrey W. Beardsall, Jeremy E. Broome, Michael J. Dadswell

**Affiliations:** Biology Department, Acadia University, Wolfville, Nova Scotia, Canada; Virginia Commonwealth University, UNITED STATES

## Abstract

In the Bay of Fundy, Atlantic sturgeon from endangered and threatened populations in the USA and Canada migrate through Minas Passage to enter and leave Minas Basin. A total of 132 sub-adult and adult Atlantic sturgeon were tagged in Minas Basin during the summers of 2010–2014 using pressure measuring, uniquely coded, acoustic transmitters with a four or eight year life span. The aim of this study was to examine spatial and seasonal distribution of sturgeon in Minas Passage during 2010–2014 and test the hypothesis that, when present, Atlantic sturgeon were evenly distributed from north to south across Minas Passage. This information is important as tidal energy extraction using in-stream, hydrokinetic turbines is planned for only the northern portion of Minas Passage. Electronic tracking data from a total of 740 sturgeon days over four years demonstrated that Atlantic sturgeon used the southern portion of Minas Passage significantly more than the northern portion. Sturgeon moved through Minas Passage at depths mostly between 15 and 45 m (n = 10,116; mean = 31.47 m; SD = 14.88). Sturgeon mean swimming depth was not significantly related to bottom depth and in deeper regions they swam pelagically. Sturgeon predominately migrated inward through Minas Passage during spring, and outward during late summer-autumn. Sturgeon were not observed in Minas Passage during winter 2012–2013 when monitoring receivers were present. This information will enable the estimation of encounters of Atlantic sturgeon with in-stream hydrokinetic turbines.

## Introduction

Many coastal regions globally are being examined for the potential extraction of tidal energy to reduce dependence on fossil fuels. Pilot projects to determine the viability of tidal energy extraction using in-stream, hydrokinetic turbines are underway with the first test center based in Orkney, Scotland operational since May 2005 [1]. Other experimental deployments of in-stream tidal turbines have occurred in Minas Passage, NS, Canada in 2009 [2] and Cobscook Bay, ME, USA in 2012 [3]. Project viability includes aspects of both potential for [4,5] and cost of, energy extraction, and identification of possible negative effects on resident and migratory organisms [6,7,8,9,3]. The introduction of tidal turbines to macro-tidal estuaries may cause widespread effects on populations of fishes and marine mammals resulting in significant declines in abundance [10]. The effect of large turbines (> 5 m diameter) is predicted to be most damaging for larger fishes from vulnerable populations [8]. The Bay of Fundy on Canada’s east coast is a macro-tidal environment where interactions between large migratory fish resources and a barrage-style tidal power turbines at Annapolis Royal have been observed [11,12].

Minas Basin at the head of the inner Bay of Fundy has the largest tidal range in the world (16+ m; [13]). Minas Passage, the entrance to Minas Basin, is the site of a developing tidal energy test centre with multiple experimental turbine deployments planned for the northern portion of Minas Passage in 2016–2018 (Fundy Ocean Research Centre for Energy, www.fundyforce.ca).

There are large migrations of fishes that enter and exit Minas Basin via Minas Passage on a seasonal basis to feed and/or reproduce [14,15,16,17,18,19]. Many at-risk species (as designated by the Committee On the Status of Endangered Wildlife In Canada [COSEWIC]) occupy habitat in Minas Basin and Minas Passage and include endangered Porbeagle shark (*Lamna nasus*), inner Bay of Fundy Atlantic salmon (*Salmon salar*), and Striped bass (*Morone saxatilis*); threatened American eel (*Anguilla rostrata*) and Atlantic sturgeon (*Acipenser oxyrhinchus*).

Atlantic sturgeon are large, anadromous fish that range from Ungava Bay, Canada [20] to northern South America [21]. Atlantic sturgeon are estuarine dependant during several phases of their life cycle [12,22,19] which is common for many species of sturgeon in North America including shortnose sturgeon (*Acipenser brevirostrum*) and green sturgeon (*Acipenser medirostris*) [23,24,25]. In the Canadian portion of their range Atlantic sturgeon juveniles migrate from natal estuaries at approximately 10 years of age [12] and spend 10 to 15 years as sub-adult oceanic residents with summer feeding [19] and overwintering phases [26]. Atlantic sturgeon males mature at ~20 y and females at ~25 y and when ripe return to their natal rivers to reproduce [12].

Atlantic sturgeon are demersal feeders [19,27] and associate with the substrate in shelf areas occupying mostly shallow depths from 6 to 81 m in coastal regions [22,26]. Atlantic sturgeon distribution and movement in the ocean is poorly known [28].

In many regions of eastern North America, Atlantic sturgeon populations were severely overexploited [29] and their spawning rivers blocked by hydroelectric dams and/or badly polluted [12]. In 1979, Atlantic sturgeon received an Appendix II listing by the Convention on International Trade in Endangered Species. By 1998, all USA Atlantic sturgeon fisheries were closed [30]. The National Oceanic and Atmospheric Administration and the U.S. Fish and Wildlife Service listed four of the five Distinct Population Segments (DPSs) in the USA as endangered, the New York Bight, Chesapeake Bay, Carolina, and South Atlantic [31]. The Gulf of Maine DPS was listed as threatened [31]. In Canada, the Saint John River, NB, and the St. Lawrence River, QC, have Atlantic sturgeon populations that support small directed fisheries [12,32].

Atlantic sturgeon that migrate into and out of Minas Basin through Minas Passage, are from both Canadian and USA stocks [33]. Genetic analysis indicated that ~60% of the sturgeon sampled in Minas Basin in 2007–2009 were from the Saint John River stock, 32–34% from the Kennebec River stock, and 1–2% from the Hudson River stock and 2% of unidentified origin [33]. As a result, the operation of in-stream hydrokinetic turbines in the northern portion of Minas Passage presents potential risks to Atlantic sturgeon from listed endangered and threatened DPSs in the USA and a designated threatened population in Canada [34,35].

Little is known about how Atlantic sturgeon use Minas Passage, except that they must traverse the passage when entering and exiting Minas Basin since they do not remain in the Basin during winter [19]. We used acoustic tagging technology to test the hypothesis that migrating Atlantic sturgeon are evenly distributed from north-to-south across Minas Passage, also, we characterized Atlantic sturgeon use of Minas Passage by examining their depth distribution, and seasonality of occurrence. This information is central to the prediction of possible interactions of Atlantic sturgeon with planned in-stream, hydrokinetic turbines and will inform turbine engineers and industry regulators of spatially and temporally important characteristics of sturgeon movement in Minas Passage.

## Materials and Methods

### Study Site

**Fig 1:**
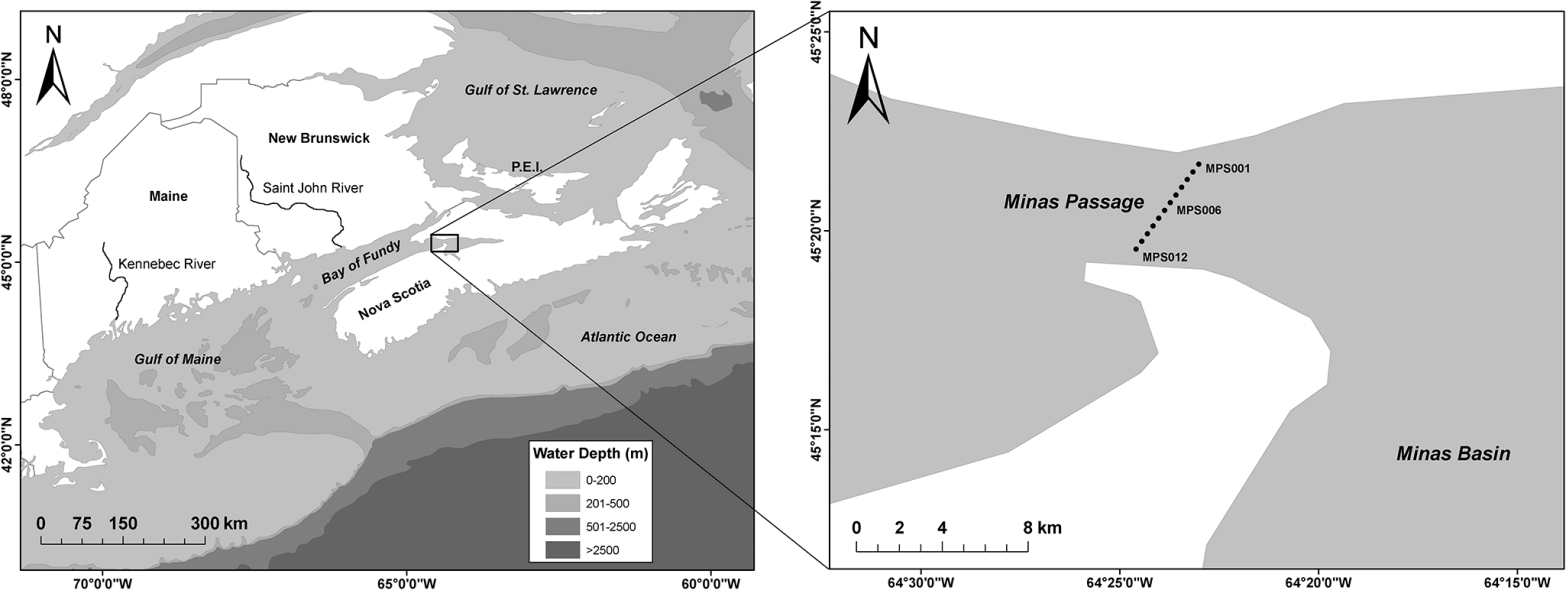
The Minas Basin is the north-eastern portion of the inner Bay of Fundy (A); Minas Passage, links Minas Basin to the rest of the Bay of Fundy (B).

Minas Passage connects Minas Basin to the outer Bay of Fundy (Fig 1). The Passage is 5 km wide, 11 km long with a maximum depth of 115 m [13]. Up to 14 billion tonnes of water (http://fundyforce.ca/) transit Minas Passage during each phase of the semi-diurnal tides. Tidal current speeds are variable and peak at approximately 6 ms^-1^ on spring tides. On the flood tide an eddy forms in the southern portion of the Passage and faster tidal speeds are reached in the middle and north of the Passage [36].

Minas Basin is a shallow (<25 m), macro-tidal, estuarine embayment with an average tidal amplitude of 11.5 m [13]. Extreme tides along with shallow bathymetric gradients create a 1–2 km wide intertidal zone in Minas Basin with variable sediment composition where a summer feeding aggregation of Atlantic sturgeon occurs [37,27].

### Receiver Infrastructure

An acoustic hydrophone array was deployed by the Ocean Tracking Network (OTN) in collaboration with Acadia University in Minas Passage during 2010–2014 (Fig 1). Moorings contained a VEMCO VR2W omnidirectional hydrophone receiver that recorded date and time, unique transmitter ID, and sensor information (i.e. depth) for transmitters within the detection radius of the receiver. Custom modified A2 Model SUB streamlined instrument floats (Open Seas Instrumentation, Musquodoboit Harbour, NS) were used to house VR2W receivers and were connected to the fiberglass strong-back of a Teledyne Benthos 875-TD acoustic release (Teledyne-Benthos, North Falmouth, Mass., USA). Mooring anchor and chain weight was 200–225 kg [38].

### Range Test and Detection Probability

Detection ranges in Minas Passage are greater for ebb tide as opposed to flood tide [36]. A range detection experiment was performed by OTN in Minas Passage, at the Minas Passage Line (Fig 1). Sanderson (unpublished data) computed the probability that an acoustic tag with signal strength 158 dB and nominal delay of 75 s (tags used in this study) would be detected at a given current velocity at least once as it passed a receiver. The current velocity is positive on the flood tide and negative on the ebb tide.

**Fig 2:**
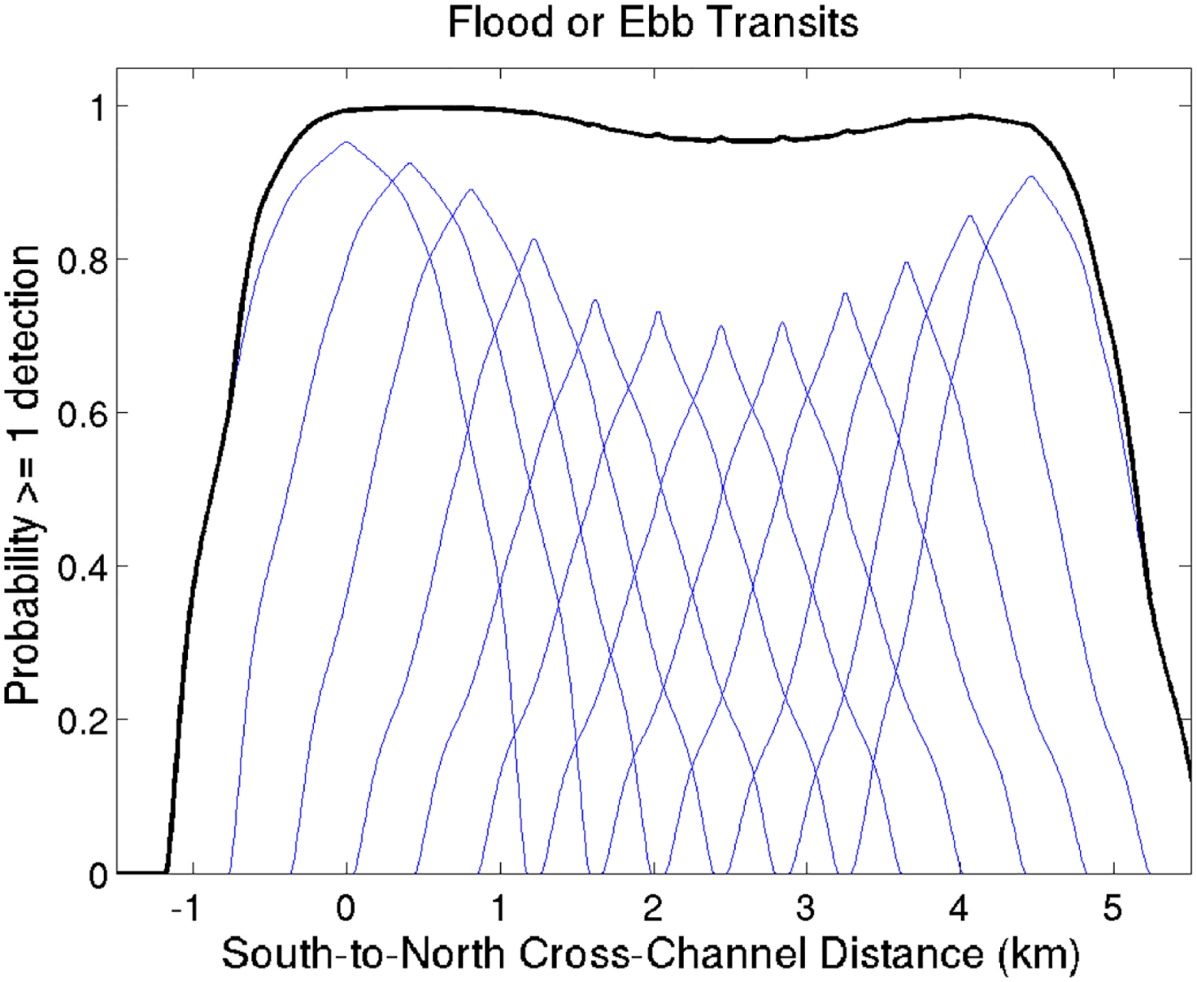
Results of OTN range test experiment calculated by Sanderson et al. (in preparation). Probability of detection of Atlantic sturgeon tagged with V16 acoustic tags (158 db, 75 sec average transmission interval) passing the OTN Minas Passage Line. Individual receivers are represented by blue lines with left hand side representing the south (first blue peak = receiver MPS012) and right side (last blue peak = receiver MPS001). Solid black line represents the probability of a tagged sturgeon being detected by at least one of the receivers. Probability of being detected by at least one receiver is nearly identical for the north and south sections of the line, with a small reduction in probability of detection in the middle.

A time series of currents was obtained by fitting tidal harmonics to the output from a hydrodynamic model for the Bay of Fundy (Karsten et al. unpublished data). These currents and closest approach to the receiver were then used to determine the detection efficiencies for acoustically-tagged fish that pass a receiver on ebb or flood tides. By appropriately combining probabilities of all 12 receivers that are spaced along the MPS line, a probability was obtained that a tagged fish will be detected at least once by at least one receiver as it passes the OTN line of receivers (Fig 2).

### Tagging and Tracking

Fishing was performed under the Department of Fisheries and Oceans Scientific License to Fish #322595. All sampling and experimental procedures were reviewed and approved by the Acadia Animal Care Committee (protocol #07–11). Atlantic sturgeon were captured in Minas Basin by otter-trawl (13 m vessel), and by intertidal fishing weirs during May to August of 2010, 2011, 2012 and 2014 [39,27]. The trawl was a 24 m-box trawl with a mesh size of 14 cm and a tickle chain with 200 kg metal Bison doors. The trawl was towed at 5.5 km hour^-1^. Tow times ranged between 15 and 150 minutes. The two, one km long, intertidal weirs that were used to collect sturgeon were made of wooden posts spaced 1 m apart driven into the sediment of the intertidal zone. Commercial netting with a 2.5 cm mesh size was used to line the weir. Weirs were V-shaped with the trap end pointed offshore. A pool was formed in the trap end where fish accumulate. Sturgeon were removed from the trap end, tagged and then returned to a pool to await the rising tide.

To facilitate transmitter insertion a large PVC cradle capped on one end was tipped at a 45° angle to allow water to pool. A stock solution of 10 mg/L of MS222 was mixed with 20 L of fresh seawater (anesthetic solution of MS222 = 0.5 mg/L) in the PVC cradle. Atlantic sturgeon were placed dorsal side up with their head and gills fully submerged in the anaesthesia bath until opercular beats were slowed and they were unresponsive to gentle stimulus such as a tail grab. Anaesthetised sturgeon were removed from the bath and placed ventral side up on a moistened tarpaulin. A 3–4 cm incision was made on the ventral surface on either side of the *linea alba*; generally anterior to the pelvic girdle. Transmitters (V16P-6x-A69-9002, 16 mm x 98 mm, depth Range 136 m (accuracy ±6.8 m, resolution 0.6 m), estimated tag life was 1287 days for tags deployed in 2010, 2011 and 2013 and 2751 days for tags deployed in 2014, Vemco Inc., Nova Scotia; Table 1) were inserted into the abdominal cavity and pushed approximately 4 cm anteriorly.

**Table 1:** Summary of coded acoustic tag models used during Atlantic sturgeon tagging project in Minas Basin during the summers of 2010, 2011, 2012 and 2014 (n = 132). All models supplied by Vemco Inc., Halifax, NS, Canada.

Tag model	Year	Number used during study	Dimensions (mm)	Weight in air (g)	Battery life(days)	Power output (dB)
V16-6x	2010	15	16 x 95	34	1633	158
	2012	20				
V16P-6x^a^	2010	10	16 x 98	36	1287+	158
	2011	53				
	2012	19				
	2014	10				
V16TP-6x^b^	2010	5	16 x 98	36	1609	158

^a^tag includes environmental sensor (pressure)

^b^tag includes environmental sensors (pressure and temperature)

Two horizontal mattress sutures, using sterile absorbable 1/0 Ethilon monofilament nylon sutures with a reverse cutting edge needle (Johnson and Johnson, Ontario), were used to close the incision site. All equipment, including transmitters were disinfected prior to surgery using a 10% Betadine solution, followed by a saline rinse. Surgeries lasted 5–10 minutes excluding anaesthesia and recovery time. Post-surgery, sturgeon were held in a recovery tank (on the trawl) and allowed sufficient time to regain equilibrium and for their condition to be monitored before being released near the capture site. All sturgeon were also marked with an external numbered, yellow, Floy FT-1-94 spaghetti dart tag (FLOY TAG Inc., Seattle, WA, USA) placed under the dorsal fin and an internal Passive Integrated Transponder tag placed under the scute anterior of the dorsal fin (Oregon RFID, Portland, OR, USA) for identification purposes. Sturgeon fork length (FL) was measured to the nearest cm, weighed to the nearest kg and sampled for DNA by taking a fin clip prior to release.

### Analysis

VR2W receiver data were downloaded and initially processed using Vemco User Environment (VUE) software twice a year. To remove behavioural bias in the detection data, we calculated “sturgeon days” for the receivers. A sturgeon day was defined as the receiver with the largest number of detections for a single tagged sturgeon, during a single day.

We used an ANOVA and post hoc Tukey test for sturgeon-day data to test the hypothesis that Atlantic sturgeon are equally distributed from north-to-south when occupying habitat in Minas Passage. We used regression analysis to test the hypothesis that receiver bottom depth and sturgeon swimming depth were not related, in which case sturgeon were swimming pelagically through Minas Passage.

## Results

In total, 132 Atlantic sturgeon were captured by otter trawl or brush weir in Minas Basin, acoustically tagged and released. Numbers of sturgeon tagged per year were 30, 53, 39 and 10 in 2010, 2011, 2012 and 2014, respectively. Acoustic receivers positioned inside Minas Basin detected these tagged sturgeon during the summer of their tagging and survival was excellent [97%, 39]. Fork length of the Atlantic sturgeon captured, measured and tagged in Minas Basin (Mean = 135.8 cm FL, SD = 20.3) indicated that most sturgeon were sub-adults. Sex could not be determined.

**Fig 3:**
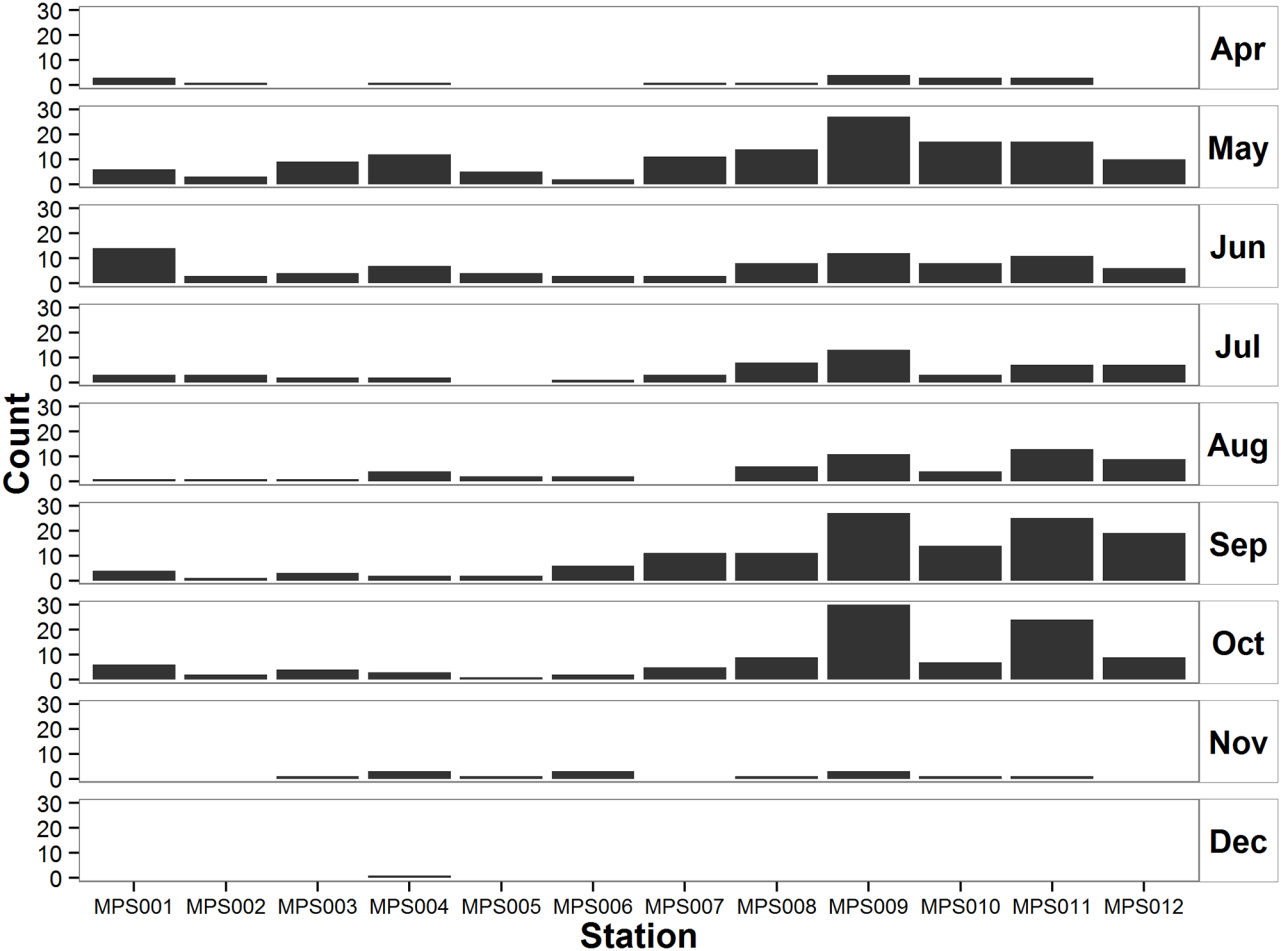
Sturgeon day distribution for acoustically tagged Atlantic sturgeon recorded on the Ocean Tracking Network Minas Passage Line, Minas Passage, Nova Scotia, Canada, totals for years 2010–2014.

A mean of 67% of total tagged Atlantic sturgeon were detected leaving Minas Basin via the Minas Passage in the first autumn after tagging and a large portion of these were detected again in Minas Passage in subsequent years (Range 46–83%; Table 2). Sturgeon north-to-south distribution in Minas Passage over the four years of the study indicated that there were significant differences in the use of different regions by sturgeon (ANOVA; *p* < 0.001, *df* = 83). A Tukey Multiple Comparison test indicated that areas around southern receiver stations MPS008, MPS009, MPS010, MPS011 and MPS012 were used significantly more by sturgeon than areas around northern receivers MPS002, MPS003, MPS004, MPS005, MPS006 and MPS007 (p < 0.05; Fig 3) with the exception of northern receiver MPS001 which recorded high use during May-June when sturgeon were entering the Basin (Fig 3). It should be noted that MPS004 was not recovered in autumn 2010.

**Table 2:** Summary of yearly detections of acoustically tagged Atlantic sturgeon that occurred in Minas Passage during 2010–2014 (n = 132).

Year	Total	Number	Number	Number	Number	Number
Tagged	Tagged	2010	2011	2012	2013	2014
**2010**	30	21	25	20	15	14
**2011**	53	-	42	27	27	30
**2012**	39	-	-	23	18	19
**2013**	0	NA	NA	NA	NA	NA
**2014**	10	-	-	-	-	6

**Fig 4:**
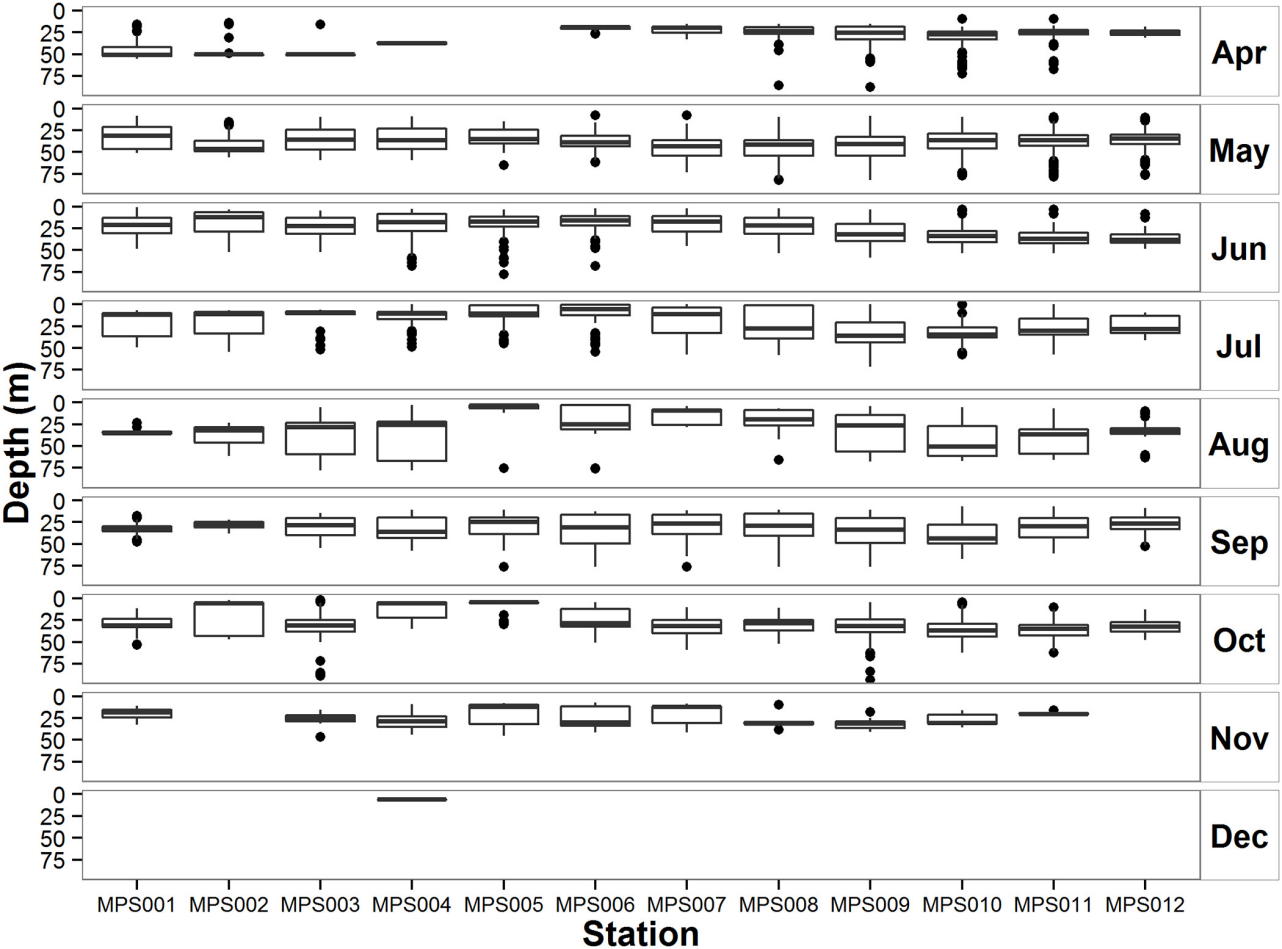
Depth box plots (Line = median, box = 25 and 75% quartiles, whiskers = SD and dots = outliers) distribution for acoustically tagged Atlantic sturgeon recorded on the Ocean Tracking Network, Minas Passage Line, Minas Passage, Nova Scotia, Canada, totals for years 2010–2014.

Depth distribution of tagged sturgeon passing the OTN Minas Passage Line was examined for 2010–2014 (Fig 4). Receivers were placed at the same depth and position each year which permitted an extensive examination of the depth distribution over multiple years. Depth detections obtained from Minas Passage during the five year period indicated that sturgeon mean depth was 31.47 m (SD = 14.88; n = 10,116), with the majority of detections in the 15 to 45 m depth range. Mean sturgeon swimming depth and receiver depth were not significantly related (df = 11; p = 0.779, R^2^ = 0.008) indicating that sturgeon were swimming pelagically through Minas Passage.

## Discussion

Atlantic sturgeon, originating from the Saint John River, New Brunswick, and smaller numbers from DPSs listed as threatened and endangered in the USA migrate through Minas Passage and into Minas Basin each spring beginning in April. Atlantic sturgeon were present in Minas Passage periodically throughout the summer, and then migrated out of Minas Basin through Minas Passage mostly in late September and early October. No tagged sturgeon were detected in Minas Passage during the winter months of 2012–2013 when hydro-acoustic receivers were present. Using pop-up archival satellite tags, researchers have found that the minimum and maximum marine water temperature experienced by tagged Atlantic sturgeon in shelf waters off the Eastern seaboard of the USA and Canada were 4.9°C (January and February; [26]) and 23.9°C (August; [22]). Water temperatures in Minas Basin are very low (sub-zero) during winter as water flows over the large, usually frozen, intertidal zone where large buoyant ice blocks form and move into deeper areas of Minas Basin [40]. It is very unlikely that Atlantic sturgeon remain in Minas Basin during winter because of the extremely low water temperatures.

Sturgeon used the southern portion of Minas Passage significantly more than the northern portion. There are slower current speeds in the southern portion of the passage due to an eddy that forms on the flooding tide [41]. As sturgeon are poor swimmers, swimming in the slower currents of the eddy may facilitate movement, particularly at time of high current speeds such as spring tides. Range test data indicates that on the OTN line acoustic receiver range in the southern portion and northern portion of Minas Passage are very similar, with slightly reduced reception in the middle of Minas Passage, therefore, it is unlikely that differences in receiver range are responsible for significantly more detections being logged in the south compared to the north. The pattern shown by the sturgeon is different than the distribution of acoustically tagged striped bass recorded by the same hydro-acoustic receivers, where striped bass were present more in the middle and northern portions of Minas Passage during winter [42]. The proposed deployment location of tidal power turbines is planned for the northern portion of Minas Passage (www.fundyforce.ca), and although sturgeon were occasionally present on the north side of the Passage, the overlap of turbines and sturgeon will be less than if the sturgeon were equally distributed across Minas Passage.

Atlantic sturgeon demonstrated a high annual rate of return to Minas Basin. Sturgeon spend the summer in Minas Basin feeding [19,27] and then likely overwinter in areas off the Saint John River [26]. As the sturgeon must move through Minas Passage between their summer feeding area, and overwintering area, annual and inter-annual cumulative effects of potential turbine interaction must be considered.

Atlantic sturgeon moved pelagically through Minas Passage. Atlantic sturgeon breach [43] so they are known to spend short periods of time in the water column, not associated with the substrate; however, this is the first report of Atlantic sturgeon migrating pelagically through an area. It is unclear whether pelagic swimming is common for sturgeon, or if it is a special strategy to deal with macro-tidal environments. Atlantic sturgeon demonstrated a narrow preference for depths centred around 30 m over the range of depths available in Minas Passage. We know the bottom depth of the receiver but the exact position of the tagged fish is not known, other than it is within range of the receiver, so some variability was introduced into the analysis; however, there was clearly not a significant relationship between swimming depth and bottom depth. Atlantic sturgeon prefer shallow near shore depths in the marine environment [22] but few observations have been made from offshore areas. In areas of in-stream turbine deployment in Minas Passage, depths occupied by turbines are likely to overlap with the depths occupied by Atlantic sturgeon.

The potential for temporal and spatial overlap between Atlantic sturgeon and tidal turbines in Minas Passage is concerning. Sturgeon are poor swimmers [44,45] with slow swimming speeds of < 0.2 m/s [46]. The ability of a sturgeon to avoid a turbine in fast water flow may be low. Fishes have shown avoidance of small turbines in low current speed environments [7], however, avoidance ability has not been examined at current speeds > 2.8 m/s [3]. For large turbines (> 5 m diameter) there is a greater probability of collision for individual (non-schooling) fishes because rotor detection and avoidance may be difficult [3]. Turbine developers must take these risk factors into account when attempting to mitigate possible effects of in-stream tidal turbine operation on Atlantic sturgeon.
